# A review on the role of DANCR in the carcinogenesis

**DOI:** 10.1186/s12935-022-02612-z

**Published:** 2022-05-19

**Authors:** Soudeh Ghafouri-Fard, Tayyebeh Khoshbakht, Bashdar Mahmud Hussen, Aria Baniahmad, Mohammad Taheri, Mohammad Samadian

**Affiliations:** 1 Department of Medical Genetics, School of Medicine, Shahid Beheshti University of Medical Sciences, Tehran, Iran; 2 Phytochemistry Research Center, Shahid Beheshti University of Medical Sciences, Tehran, Iran; 3 Department of Pharmacognosy, College of Pharmacy, Hawler Medical University, Erbil, Kurdistan Region Iraq; 4 Center of Research and Strategic Studies, Lebanese French University, Erbil, Kurdistan Region Iraq; 5 Institute of Human Genetics, Jena University Hospital, Jena, Germany; 6 Urology and Nephrology Research Center, Shahid Beheshti University of Medical Sciences, Tehran, Iran; 7 Skull Base Research Center, Loghman Hakim Hospital, Shahid Beheshti University of Medical Sciences, Tehran, Iran

**Keywords:** DANCR, lncRNA, Cancer

## Abstract

*DANCR* is an RNA gene located on chr4. This gene has several splice variants. Up-regulation of DANCR has been reported in many types of cancers. This lncRNA is mainly located in the cytoplasm and regulates genes expression at post-transcriptional level. In fact, it acts as a molecular sponge for a variety of miRNAs, including miR-874-3P, miR-335, miR-149, miR-4319, miR-758-3p, miR-216a-5p, miR-874-3p, miR-33a-5p, miR-335-5p, miR-145-3p, miR-665, miR-345-5p and miR-125b-5p. DANCR also regulates activity of PI3K/AKT/NF-κB, Wnt/β-catenin, ERK/SMAD, MAPK, IL-6/JAK1/STAT3, Smad2/3, p53, FAK/PI3K/AKT/GSK3β/Snail pathways. In the current narrative review article, we summarize the roles of DANCR in the carcinogenesis, with an especial emphasis on its role in the development of osteosarcoma and lung, liver, pancreatic and colorectal cancers.

## Introduction

*DANCR* (Differentiation Antagonizing Non-Protein Coding RNA) is an RNA gene located on chr4: 52,712,257–52,723,623, plus strand. It has a size of 11,367 bases. This gene has 14 splice variants with sizes ranging from 272 bp (DANCR-207) to 6065 bp (DANCR-203), all of them being categorized as long non-coding RNA (lncRNA). This lncRNA has been regarded as a cancer-associated lncRNA, since its up-regulation has been reported in several cancer types in association with enhancement of cell proliferation and malignant properties [1]. DANCR regulates gene expression at post-transcriptional level [1]. Based on the findings obtained from RNA fluorescence in situ hybridization and expression assays in the cellular fractions, DANCR has been found to be primarily located in the cytoplasm [2]. In the current narrative review article, we summarize the roles of DANCR in the carcinogenesis, with an especial emphasis on its role in the development of osteosarcoma and lung, liver, pancreatic and colorectal cancers.

## Cell line studies

Up-regulation of DANCR has been shown to upsurge proliferation, migratory propensity, and invasiveness of osteosarcoma cells. From a functional aspect, DANCR promotes progression of osteosarcoma through induction of cancer stem cells properties. DANCR up-regulates expression of AXL through sequestering miR-33a-5p. Further, DANCR enhances activity of AXL/Akt pathway. Cumulatively, DANCR is an important regulator of osteosarcoma progression [2]. Another study in osteosarcoma cells has indicated that inhibition of DANCR leads to decrease in ROCK1-mediated proliferation and metastasis. Mechanistically, DANCR regulates expression of ROCK1 through sequestering miR-335-5p and miR-1972 [3]. Other studies have revealed the impacts of DANCR/miR-149/MSI2 axis [4] and DANCR/miR-216a-5p/SOX5 [5] axes in the pathoetiology of osteosarcoma. Moreover, METTL3 has been shown to contribute in this type of cancer through enhancement of stability of DANCR transcripts through m6A modification [6].

In bladder cancer cells, DANCR silencing has inhibited proliferation, migratory potential and invasion. DANCR has been shown to target miR-335/VEGF-C. miR-335 mimics could promote proliferation and invasive properties bladder cancer cells. In contrast, up-regulation of DANCR removes the effect of miR-335 mimics on these cells [7]. In addition, DANCR enhances metastatic and proliferative abilities of bladder cancer cells through increasing IL-11-STAT3 signals and CCND1 levels [8]. Finally, miR-149/MSI2 has been identified as another route of participation of DANCR in progression of bladder cancer [9].

**Fig. 1:**
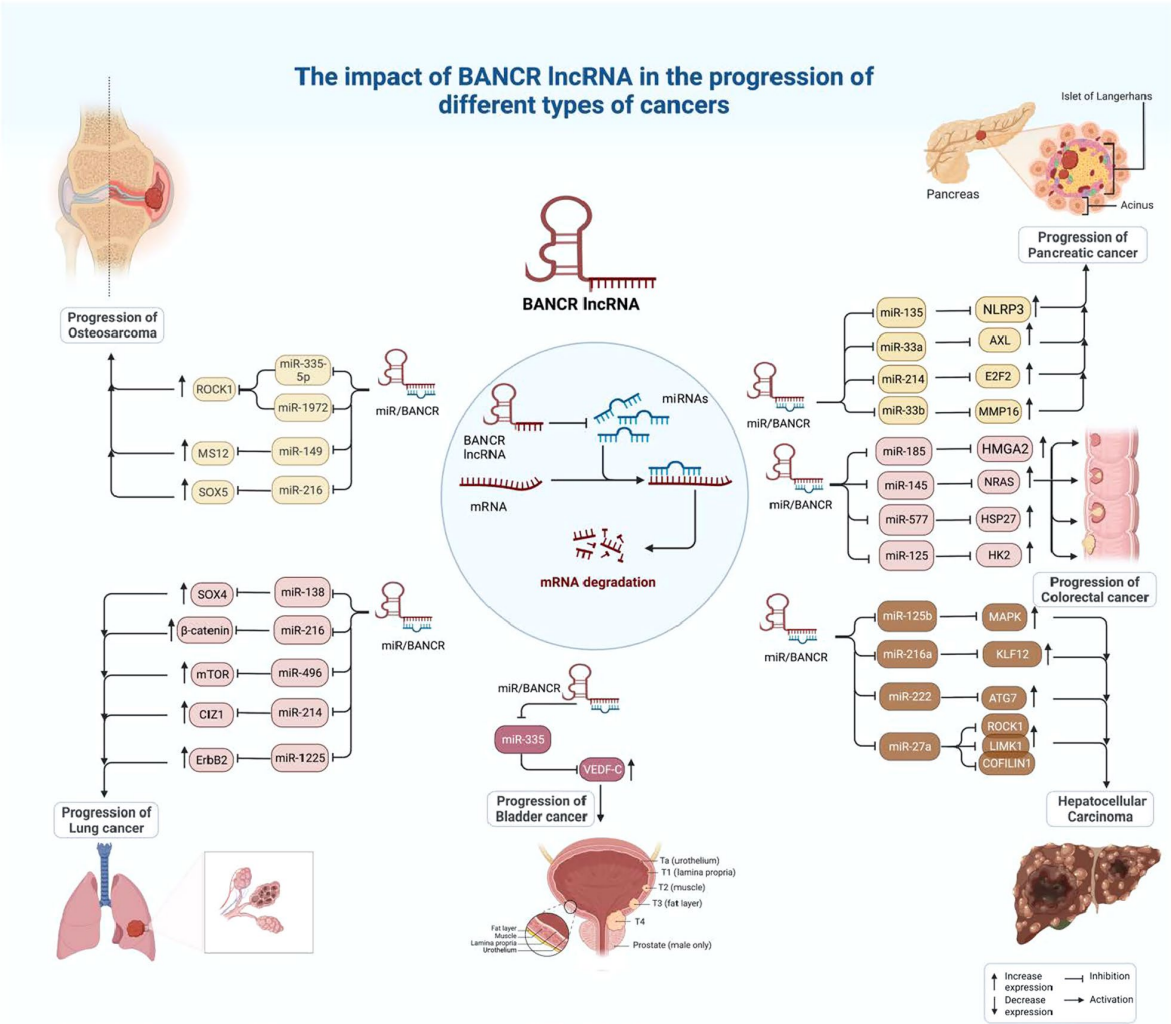
Oncogenic role of DANCR in osteosarcoma, lung cancer, liver cancer, colorectal cancer, bladder cancer, and pancreatic cancer

In lung cancer cells, DANCR expression levels have been negatively correlated with levels of miR-216a [10]. Another study has identified the impact of DANCR/miR-1225-3p/ErbB2 axis in the regulation of metastasis of lung cancer cells [11]. Moreover, DANCR participates in the progression of this type of cancer through sequestering miR‐496 and further modulating expression of mTOR [12]. DANCR can also regulate miR-214-5p/CIZ1 axis [13]. Moreover, invasive properties of lung cancer cells are regulated by DANCR through suppression of miR-216 and subsequent activation of Wnt/β-Catenin signals [14]. Figure 1 shows roles of DANCR in osteosarcoma, lung cancer, liver cancer, colorectal cancer, bladder cancer, and pancreatic cancer.

Hepatocellular carcinoma is another type of cancer in which DANCR has an important effect. Up-regulation of DANCR in these cells has been associated with down-regulation of miR-125b-5p. DANCR silencing or miR-125b-5p mimics could reduce cell cycle progression in HepG2 or Huh-7 cells, while promoting cell apoptosis. Both interventions could also inhibit migratory potential and invasiveness of these cells. Mechanistically, DANCR facilitates progression of this cancer through sponging miR-125b-5p and activating MAPK pathway [15]. DANCR could also contribute in the liver carcinogenesis through sponging miR‐216a‐5p and surging expression of KLF12 [16]. Another study in hepatocellular carcinoma cells has shown over-expression of DANCR and ATG7, and down-regulation of miR-222-3p. Besides, DANCR silencing has intimidated proliferation and autophagy of these cells. Mechanistically, DANCR induces proliferation, colony construction and autophagy of these cells through enhancing expression of ATG7 and decreasing expression of miR-222-3p [17]. Notably, DANCR can also affect response of hepatocellular carcinoma cells to sorafenib through enhancing activity of IL-6/STAT3 signals [18]. This lncRNA can also affect stemness and epithelial–mesenchymal transition (EMT) through modulating expression of CTNNB1 [19] and regulation of activity of ROCK1/LIMK1/COFILIN1 pathway [20], respectively.

In colorectal cancer cells, DANCR has been shown to affect activity of miR-125b-5p/HK2 axis to induce resistance to cisplatin through induction of anaerobic glycolysis [21]. In addition, DANCR/miR-518a-3p/MDMA axis has been identified as an imperative regulator of growth and malignant behavior of these malignant cells [22]. Most notably, the interaction between DANCR and the important oncogenic lncRNA MALAT1 has been found to induce resistance to doxorubicin-associated apoptosis in colorectal cancer cells [23].

In pancreatic cancer cells, DANCR regulates expression of miR-33b to promote proliferation and metastatic abilities [24]. Moreover, the invasive properties of these cells are regulated by DANCR/miR-214-5p/E2F2 [25] and DANCR/miR-135a/NLRP37 [26] axes. Figure 1 shows oncogenic roles of DANCR in osteosarcoma, lung cancer, liver cancer, colorectal cancer, bladder cancer, and pancreatic cancer. Expression of DANCR has been found to be increased in triple negative breast cancer cell lines. Notably, DANCR silencing has led to suppression of proliferation of these cells. Functional studies have detected that DANCR binding with RXRA enhances phosphorylation of this protein on its serine 49/78 via GSK3β, which subsequently leads to activation of PIK3CA transcription, and induction of PI3K/AKT signals [27]. Another study has shown over-expression of DANCR and VAPB in breast cancer cells, parallel with down-regulation of miR-4319. DANCR silencing not only has stalled proliferation, migratory potential, and invasiveness of breast cancer cells, but also has induced their apoptosis. These effects have been found to be mediated through regulation of miR-4319. This study has revealed the importance of DANCR/miR-4319/VAPB axis in development of this cancer [28]. Another mechanism of involvement of DANCR in the pathogenesis of breast cancer is mediated through enhancement of the EZH2 binding to the promoter of SOCS3, which results in suppression of expression of SOCS3. Up-regulation of SOCS3 or suppression of EZH2 has led to reversion of malignant features stimulated by DANCR [29].

Expression of DANCR has been found to be high in cisplatin-resistant gastric cancer cells. However, siRNA-mediated silencing of this lncRNA in SGC7901/DDP and BGC823/DDP cells has led to significant decrease in their survival and induction of apoptosis. Furthermore, DANCR up-regulation could up-regulate expression levels of MDR1 and MRP1 in cisplatin resistant gastric cancer cells [30]. Another study in gastric cancer cells has shown that KLF5 activates DANCR transcription. DANCR could act as a molecular sponge for miR-194 to suppress its expression and increase expression of AKT2, thus promoting gastric carcinogenesis through inhibition of autophagy [31]. Moreover, expression of DANCR in gastric cancer can be induced by SALL4 [32].

Table 1 summarizes the molecular axes mediating the effects of DANCR in the carcinogenesis, based on the results of in vitro studies.

**Table 1:** Expression of DANCR in cell lines

Tumor type	Interactions	Cell line	Function	References
Acute myeloid leukemia	miR-874-3P/ATG16L1 axis	HL60, U937, and KG1a	DANCR is involved in Ara‐C resistance and promotes autophagy in HL60 cells via regulating ATG16L1	[33]
Bladder cancer	miR-335/VEGF-C axis	SW780, 5637, T24, UM-UC-3, SV-HUC-1, and T24	∆ DANCR: ↓proliferation, migration, invasion and lymphatic metastases	[7]
	IL-11-STAT3 signaling and CCND1	UM-UC-3, T24 and 293T	DANCR was found to promote bladder cancer progression	[8]
	miR-149/MSI2 axis	5637, SW780, UM-UC-3, T24 and SV-HUC-1	∆ DANCR: ↓ proliferation, migration, invasion and EMT process	[9]
Breast cancer	PI3K/AKT signaling	BT549, MCF7, T47D, MDA-MB-231, MDA-MB-453, and MDA-MB-468 and MCF10A	∆ DANCR: ↓ proliferation and tumor growth DANCR is involved in enhancing PI3K/AKT signaling by binding with RXRA and increasing its serine 49/78 phosphorylation	[27]
	miR-4319/VAPB axis	MCF-10A, MCF-7 and HCC38	∆ DANCR: ↓ proliferation, migration, invasion, and ↑ apoptosis	[28]
	EZH2, SOCS3	MCF10A, MCF7, T47D, MDA‐MB‐231, and MDA‐MB‐468	∆ DANCR: ↓ viability, migration and invasion DANCR epigenetically inhibits SOCS3 expression	[29]
	miR-758-3p-PAX6 axis	HCC1937, 1590, ZR-75-30, MDA-MB-468 and MCF-10A	∆ DANCR: ↓ proliferation and ↑ apoptosis	[34]
	miR-216a-5p	MCF-7, MDA-MB-231 and MCF-10A	∆ DANCR: ↓ proliferation, migration, and invasion	[35]
	miR-874-3p/SOX2 axis	MCF10A, ZR751, MCF7, SKBR3, BT474, MDA-MB-231, MDA-MB-468 cells	∆ DANCR: ↓ proliferation TUFT1 induces the expression of DANCR	[36]
	EZH2, CD44 and ABCG2	Hs578Bst, MCF-7, T47D, MDA-MB-468 and MDA-MB-231	∆ DANCR: ↓ proliferation and invasion and ↑ binding of EZH2 on the promoters of CD44 and ABCG2, so reduction of expression of these genes	[37]
	PRC2, Wnt/EMT signaling	HMECs, MCF7, ZR-75–1, MDA-MB-231, Hs578T, and BT549	RGD-PEG-ECO/siDANCR nanoparticles: ↓ proliferation, invasion and migration	[38]
Cervical cancer	miR-335-5p/ROCK1 axis	aski, SW756, SiHa, C33A, HeLa, ME‐180, and End1/E6E7	∆ DANCR: ↓ proliferation, migration, and invasion	[39]
	FRAT1, FRAT2 and Wnt/β-catenin signaling pathway	HCerEpiC, HeLa, SiHa, C-33A, and ME-180	∆ DANCR: ↓ proliferation FRAT1 and FRAT2 are up-regulated by DANCR and the Wnt/β-catenin signaling pathway is activated by DANCR	[40]
	miR-145-3p/ZEB1 axis and KLF5	HeLa, SiHa, and H8	KLF5-induced up-regulation of DANCR up-regulates ZEB1 via sponging miR-145-3p to promote the progression of cervical cancer	[41]
	miR-665/TGFBR1 axis and ERK/SMAD pathway	Endl/E6E7 and H8	↑↑ miR-665 (a target of DANCR): ↓ proliferation, migration, and invasion miR-665 reduces TGFBR1 levels and inactivates ERK/SMAD pathway	[42]
	miR-345-5p/Twist1 axis	HuH28, HuCCT1, SG231, and H69	∆ DANCR: ↓ proliferation, migration, invasion, EMT and angiogenesis and ↑ apoptosis	[43]
Cholangiocarcinoma	EZH2 and FBP1	HuCCT1 and RBE	∆ DANCR: ↓ proliferation, migration DANCR could modulate the histone methylation of promoter of FBP1by binding with EZH2	[44]
	miR-125b-5p/HK2 axis	HT-29, SW620, HCT116, SW480, DLD-1, and CRL-1790	∆ DANCR: ↓ glycolysis rate and ↑ cisplatin sensitivity	[21]
Colorectal cancer	miR-518a-3p/MDM2 axis, Smad2/3 and p53	HT29, HCT116, SW116, Caco-2, and FHC	∆ DANCR: ↓ proliferation, viability, metastasis	[22]
	–	SW620, SW480, HCT116, HT29, HCT15, Caco-2, and HCoEpiC	∆ DANCR: ↓ proliferation, colony formation, and ↑ apoptosis	[45]
	MALAT1 and QK	HCT116, RKO, SW620, HT-29, and LoVo	DANCR inhibits Doxorubicin-induced apoptosis via enhancing the RNA stability of MALAT1 and interacting with QK	[23]
	miR-185-5p/HMGA2 axis	NCM460 and the CRC cell lines LoVo, SW620, SW480, and HT29	∆ DANCR: ↓ proliferation, migration, invasion and cell cycle progression, and ↑ apoptosis	[46]
	KAT6A	LOVO, SW480, HCT116, SW620, and HT29	∆ DANCR: ↓ proliferation, cell cycle progression, and tumorigenesis DANCR was found to bind with lysine acetyltransferase 6A to mediate KAT6A acetyltransferase activity	[47]
	miR-145-5p/NRAS axis	–	DANCR showed an indirect effect on NRAS expression levels via targeting miR-145-5p	[48]
	miR-577/HSP27 axis	HT29, HCT116, SW480, and LOVO and NCM460	∆ DANCR: ↓ proliferation and metastasis	[49]
	miR-214	KLE, RL95-2, ishikawa, AN3CA, and HEC-1B	∆ DANCR: ↓ proliferation and ↑ apoptosis	[50]
Endometrial carcinoma	ZNF750, and miR-4707-3p/FOXC2 axis	SHEE, KYSE140, KYSE150, KYSE180, KYSE410, KYSE510, KYSE450, Colo680N, and ECA109	Down-regulation of ZNF750 induces DANCR expression, thus inhibits miR-4707-3p to interact with FOXC2, resulting in enhanced FOXC2 signaling and angiogenesis	[51]
Esophageal squamous cell carcinoma	miR-33a-5p/ZEB1 axis	EC9706, EC109, EC1, KYSE150, and Het-1A	↑↑ miR-33a-5p (a target of DANCR): ↓ proliferation and metastasis	[52]
	–	ECA109 and TE-1	∆ DANCR: ↓ proliferation, migration, invasion, and ↑ apoptosis	[53]
Gastric cancer	MDR1 and MRP1	SGC7901 and BGC823	∆ DANCR: ↓ survival and increased apoptosis	[30]
	miR-194/AKT2 axis and	SGC7901, MGC-803, NCI-N87, and GES-1	∆ DANCR: ↓ viability, ↑ autophagy, and apoptosis KLF5 is involved in activating the transcription of DANCR	[31]
	SALL4 and β-catenin pathway	GES-1, BGC-823, MGC-803, HGC-27 and MKN-45	∆ DANCR: ↓ proliferation, migration, invasion and EMT process, ↑ cell cycle arrest and apoptosis DANCR activated by SALL4 plays its oncogenic roles via the activation of β-catenin pathway	[32]
	–	SGC7901, MGC803, and MKN-45	∆ DANCR: ↓ proliferation, and ↑ cell cycle arrest	[54]
Glioma	miR-135a-5p/BMI1 axis	LN229, U251 and NHAs	∆ DANCR: ↓ proliferation, migration and invasion	[55]
	miR-33a-5p	HEB, U87, U251, LN22 9 and T98G	∆ DANCR: ↓ proliferation, migration, and EMT process, and ↑ apoptosis	[56]
	Wnt/β-catenin signaling	U87, U251, SGC7901 and BGC823	∆ DANCR: ↓ proliferation, migration, and EMT process	[57]
	miR-33a-5p, miR-33b-5p, miR-1-3p, miR-206, and miR-613/AXL axis and PI3K/Akt/NF-κB signaling pathway	U87MG, U251MG, LN18 and U138MG	↑↑ DANCR: ↓ sensitivity of glioma cells to cisplatin ∆ DANCR: ↑ sensitivity of glioma cells to cisplatin DANCR up-regulates AXL to actives PI3K/Akt/NF-κB signaling pathway	[58]
	miR-634/RAB1A axis	U251, U118, LN229, U87MG, and NHA	∆ DANCR: ↓ proliferation and ↑ G0/G1 phase arrest	[59]
	miR-216a/LGR5, PI3K/AKT	SHG-44, U87MG, U118MG, and U251MG	∆ DANCR: ↓ proliferation, migration, invasion, angiogenesis, and ↑ phase arrest and apoptosis	[60]
	IGF2BP2, FOXO1, PID1	U251MG, LN229, LN18, T98G, and HEK293T	IGF2BP2 increases DANCR stability and decreases DANCR methylation. DANCR indices ubiquitination of FOXO1 via interacting with FOXO1. PID1 promoted by FOXO1 enhances the chemotherapy sensitivity of GBM cells	[61]
	miR-125b-5p/MAPK pathway axis	HepG2 and Huh-7 cells	∆ DANCR: ↓ migration, invasion	[15]
Hepatocellular carcinoma	miR-216a-5p/KLF12 axis	Huh7, HepG2 and LO2 cells	∆ DANCR: ↓ proliferation, migration, invasion and ↑ apoptosis	[16]
	miR-222-3p/ ATG7 axis	Bel7407, Hep3B, HepG2, Huh7, MHCC97H and LO2	∆ DANCR: ↓ proliferation and autophagy	[17]
	PSMD10-IL-6/STAT3 signaling axis	HEK-293T, Huh7 and Hep3B	DANCR promotes sorafenib resistance via PSMD10-IL-6/STAT3 signaling axis	[18]
	CTNNB1	HCC cells	DANCR is involved in stemness features of hepatocellular carcinoma by derepression of CTNNB1	[19]
	miR-27a-3p/ROCK1/LIMK1/COFILIN1 pathway axis	MHCC‐97H, Huh7, HCC‐LM3, HepG2, MHCC‐97L, Hep3B, SMMC‐7721 and LO2	∆ DANCR: ↓ proliferation, and metastasis	[20]
	β-catenin pathway	SMMC-7721 and HCCLM3	∆ DANCR: ↓ proliferation and metastasis	[62]
	miR-216a	BEAS-2B, NCI-H1299, A549, and NCI-H1975	∆ DANCR: ↓ proliferation and colony formation	[10]
Lung cancer	miR-1225-3p/ ErbB2 axis	16HBE, A549, SPCA1, H1299 and H1975	∆ DANCR: ↓ Migration and Invasion	[11]
	miR-214-5p/CIZ1 axis	16HBE, A549, SPCA1, H1299, and H358	∆ DANCR: ↓ proliferation and ↑ apoptosis	[13]
	miR-496/mTOR axis	A549, H1299, H358, (HEK) 293T cells and HBE	∆ DANCR: ↓ proliferation, migration, invasion and ↑ apoptosis	[12]
	HMGA2	16HBE, SPCA1, A549, H1299 and H1975	∆ DANCR: ↓ invasion ↑↑ DANCR: ↑ invasion via increasing HMGA2	[63]
	miR-216a and Wnt/β-catenin pathway	A549, H1975, H1755, H1944, H2087, H358, H661 and H1299	∆ DANCR: ↓ proliferation, stemness, migration, invasion	[14]
	p21	A549, H1299, H358 and BEAS-2B	∆ DANCR: ↓ proliferation, migration, invasion EMT process, ↑ apoptosis and cell cycle arrest DANCR inhibits p21 expression	[64]
	miR-138/Sox4 axis	NHBE, HEK-293T, A549, H1299, H460, SK-MES-1, and Calu-3	∆ DANCR: ↓ proliferation, migration, invasion EMT process, and ↑ apoptosis	[65]
	miR-758-3p	SPC-A, NCL-H1650, NCL-H1975, SK-MES-1, A549, NCL-H358, NCI-H1299 and 16HBE	∆ DANCR: ↓ viability, proliferation and ↑ cell cycle arrest	[66]
	–	HT-29 and FHC	∆ DANCR: ↓ proliferation, migration, invasion EMT process, and metastasis	[67]
	miR-135b-5p/KLF9 axis	MM cells	∆ DANCR: ↓ proliferation, migration, and invasion	[68]
Multiple myeloma	IL-6/JAK1/STAT3 signaling	NP460, CNE1, CNE2, HNE1, HNE2, HONE1, 5–8 F, and 6-10B	∆ DANCR: ↓ proliferation and invasion IL-6 is involved in DANCR expression upregulation via an STAT3-dependent manner DANCR interacts with STAT3 and enhances JAK1 binding to STAT3	[69]
Nasopharyngeal carcinoma	RBM3 and SOX2	C666-1, SUNE-1, HNE-1, CNE1, CNE2, and NP69	∆ DANCR: ↓ proliferation, colony formation DANCR functions as an oncogene via binding to RBM3 to stabilize SOX2 mRNA	[70]
	PTEN, AKT	5-8F, SUNE-1, C666-1, and NP69	∆ DANCR: ↓ proliferation, colony formation, and migration, and ↑ apoptosis DANCR is involved in expression of PTEN	[71]
	EZH2 and PTEN	SUNE-1 and 5-8F	∆ DANCR: ↓ cell growth and migration DANCR mediates the binding of EZH2 on PTEN promoter to down-regulate PTEN expression	[72]
	HIF-1α, NF90/NF45 complex	SUNE-1, HONE-1, CNE-1, CNE-2, HNE-1, 5-8F, 6-10B and C666-1, and S18 and S26	∆ DANCR: ↓ migration and invasion DANCR increases stability of HIF-1α mRNAs	[73]
	miR-338-3p/B4GALT3 axis	neuroblastoma cells	∆ DANCR: ↓ proliferation and ↑ apoptosis	[74]
Neuroblastoma	miR-216a-5p/Bcl-2/KLF12 axis	SCC9, SCC15, SCC25, CAL-27 and Tca8113, and NHOKs	∆ DANCR: ↓ proliferation, migration, invasion, and ↑ apoptosis	[75]
Oral squamous cell carcinoma	miR-335-5p/miR-1972/ROCK1 axis	MG-63, U2OS, MNNG/HOS, 143B and hFOB 1.19	∆ DANCR: ↓ proliferation, migration, invasion and metastasis	[3]
Osteosarcoma	miR-33a-5p/AXL axis, PI3K-Akt signaling pathway	MG63, U2OS, SaOS2, HOS, and 143B FOB, and fibroblast NIH3T3 and 293T	∆ DANCR: ↓ proliferation, migration, invasion	[2]
	miR-149/MSI2 axis	hFOB1.19 and Saos-2	∆ DANCR: ↓ proliferation, migration, invasion	[4]
	miR-216a-5p/SOX5 axis	MG-63, U2OS, 143B and hFOB 1.19	∆ DANCR: ↓ proliferation, migration, invasion and autophagy and ↑ apoptosis	[5]
	METTL3	Saos-2, SJSA-1, MG63, HOS, and U-2 OS, and hFOB 1.19	∆ DANCR: ↓ proliferation, migration, invasion METTL3 was found to regulate DANCR expression by m6A modification-mediated DANCR mRNA stability	[6]
	SP1	CAOV3, SKOV3, A2780	∆ DANCR: ↓ viability, migration and invasion SP1 could induce DANCR expression by binding to the promoter region of DANCR in ovarian cancer tissues and cells	[76]
Ovarian cancer	miR-214/TGF-ß axis	A2780 and SKOV3	∆ DANCR: ↓ viability, migration and invasion, and ↑ apoptosis	[77]
	miR-145/VEGF axis	A2780, PA‐1, SKOV3, HO8910, and HOEC	∆ DANCR: ↓ tube formation, angiogenesis, and invasion	[78]
	UPF1	IOSE-386, SKOV-3, OVCAR3, HO8910, and HEY	↑↑ DANCR: ↑ proliferation, migration via negatively regulating UPF1 level	[79]
	miR-33b/MMP16 axis	AsPC‐1, PANC‐1, CFPAC‐1, SW1990, BxPC‐3 and HPDE6‐C7	∆ DANCR: ↓ proliferation, migration, and invasion and EMT process	[24]
Pancreatic cancer	miR-214-5p/E2F2 axis	PANC-1, SW1990, CAPAN-1, BxPC-3, AsPC-1 and HPDE6-C7	∆ DANCR: ↓ growth and metastasis	[25]
	miR-33a-5p/AXL axis	Panc1, Panc28, AsPC1, MiaPaCa2 and BxPC3 and HPDE	∆ DANCR: ↓ proliferation, and colony formation	[80]
	miR-135a /NLRP3 axis	BxPC-3, MIA-PaCa-2, CFPAC-1, PANC-1, SW1990 and HPDE6-C7	∆ DANCR: ↓ proliferation and invasion	[26]
	miR-135a	RWPE-1, PC3, C4-2 and DU145	∆ DANCR: ↑ Paclitaxel Sensitivity	[81]
Prostate cancer	miR-185-5p/LASP1 axis and FAK/PI3K/AKT/GSK3β/Snail pathway	C4-2, PC3, DU145, LNCaP, 22RV1, and RWPE-1	∆ DANCR: ↓ proliferation, migration, invasion, G1-S transition and EMT process	[82]
	miR-214-5p/TGF-β axis	DU145, 22Rv1, RC-92a, PC-3M and RWPE-1	↑↑ DANCR: ↑ proliferation and migration, and ↓ apoptosis	[83]
	miR-34a-5p/JAG1 axis	DU145 and PC3	∆ DANCR: ↑ sensitivity to docetaxel	[84]
	TIMP2/3, EZH2	CWR22Rv1, PC-3, and C4-2B	∆ DANCR: ↓ migration and invasion ↑↑ DANCR: ↑ invasion and metastasis	[85]
	–	786-O and ACHN	↑↑ DANCR: ↓ proliferation, migration and invasion, and ↑ apoptosis	[86]
Renal cell carcinoma	miR-34c and miR-613/ MMP-9 axis	Weri-Rb1, Y79, SO-RB50, HXO-RB44, ARPE-19, and hTERT-RPE1	∆ DANCR: ↓ proliferation, migration, invasion, and EMT process	[87]
Retinoblastoma	miR-135a-5p/KLF8 axis and MMP-2/9	SCC9, TSCCA, TCa-8113, CAL-27 cells, and SCC9	∆ DANCR: ↓ proliferation, viability, migration and invasion	[88]
Tongue squamous cell carcinoma				

∆: knock-down or deletion, EMT: epithelial–mesenchymal transition, TNBC: Triple negative breast cancer, GBM: glioblastoma

## Animal studies

Up-regulation of DANCR in osteosarcoma cells has been shown to promote xenograft tumor growth and lung metastases [2]. Critical roles of this lncRNA in induction of metastatic pathways have also been confirmed in animal models of colon cancer [22], nasopharyngeal carcinoma [73] and prostate cancer [85]. Moreover, results of experiments in animal models of cancer have suggested the impact of DANCR in resistance to sorafenib and cisplatin in hepatocellular carcinoma [18] and colon cancer [21], respectively. Moreover, bulk of evidence from investigations in xenograft models of cancer firmly supports the role of DANCR in induction of tumor growth (Table 2).

**Table 2:** Function of DANCR in animal models

Tumor type	Results	References
Bladder cancer	∆ DANCR: ↓ tumor volume, tumor growth and metastasis	[8]
	∆ DANCR: ↓ tumor weight, and tumor growth	[9]
Breast cancer	∆ DANCR: ↓ tumor growth	[27]
	∆ DANCR: ↓ tumor growth	[29]
	∆ DANCR: ↓ tumor growth	[35]
	∆ DANCR: ↓ tumor weight, tumor volume	[36]
	∆ DANCR: ↓ tumor growth	[37]
	RGD-PEG-ECO/siDANCR nanoparticles: ↓ proliferation	[38]
Cervical cancer	∆ DANCR: ↓ tumor growth	[40]
	∆ DANCR: ↓ tumor weight, tumor volume, and tumor growth	[41]
	↑ miR-665 (a target of DANCR): ↓ tumor weight and tumor growth	[42]
Cholangiocarcinoma	∆ DANCR: ↓ tumor growth	[43]
	∆ DANCR: ↓ tumor weight and tumor growth	[44]
Colon cancer	∆ DANCR: ↓ glycolysis rate and ↑ cisplatin sensitivity	[21]
	∆ DANCR: ↓ tumor formation and metastasis	[22]
	∆ DANCR: ↓ tumor volume, and tumor growth	[45]
Colorectal cancer	↑↑ DANCR: ↑ tumor volume and tumor growth	[49]
Gastric cancer	∆ DANCR: ↓ tumor growth	[31]
	∆ DANCR: ↓ tumor weight, tumor volume, tumor size and proliferation	[32]
	↑↑ DANCR: ↑ cell growth and tumorigenicity	[54]
Glioma	∆ DANCR: ↓ tumor weight, tumor volume, and tumor growth	[55]
	∆ DANCR: ↑ apoptosis-inducing roles of cisplatin in vivo	[58]
Hepatocellular carcinoma	↑↑ DANCR: ↑ sorafenib resistance	[18]
	∆ DANCR: ↓ cell vitality, tumor shrinkage	[19]
	∆ DANCR: ↓ tumor growth and lung metastasis	[20]
	∆ DANCR: ↓ tumor growth and lung metastasis	[62]
Lung cancer	∆ DANCR: ↓ tumor growth	[10]
	∆ DANCR: ↓ tumor growth	[12]
	∆ DANCR: ↓ tumor weight, tumor volume and tumor growth	[65]
	∆ DANCR: ↓ tumor growth	[66]
	∆ DANCR: ↓ tumor volume	[67]
Nasopharyngeal carcinoma	∆ DANCR: ↓ tumor size and tumor growth	[71]
	∆ DANCR: ↓ tumor volume and tumor weight	[72]
	∆ DANCR: ↓ invasion and metastasis	[73]
Oral squamous cell carcinoma	∆ DANCR: ↓ tumor weight, tumor volume, and tumor growth	[75]
Osteosarcoma	↑↑ DANCR: ↑ tumor growth and metastasis	[3]
	∆ DANCR: ↓ tumor size and tumor volumes	[2]
	∆ DANCR: ↓ tumor growth and autophagy	[5]
	∆ METTL3: ↓ tumor volumes (DANCR could be a target of METTL3)	[6]
Ovarian cancer	∆ DANCR: ↓ tumor weight, tumor volume, and tumor growth	[78]
Pancreatic cancer	DANCR was up-regulated as pancreatic cancer progressed	[89]
	∆ DANCR: ↓ tumor growth	[25]
Prostate cancer	∆ DANCR: ↓ tumor weight, tumor volume, and tumor growth	[84]
	∆ DANCR: ↓ metastasis	[85]
Tongue squamous cell carcinoma	∆ DANCR: ↓ tumor growth and tumor formation	[88]

∆: knock-down or deletion, NOD-SCID-gamma: severe combined immunodeficient, GBM: glioblastoma

## Clinical studies

Expression of DANCR has been constantly enhanced in osteosarcoma samples, and its up-regulation has been positively associated with size of tumors and their metastatic ability. In fact, it is regarded as an independent poor prognostic factor for osteosarcoma. Besides, in patient samples, DANCR expression has been positively correlated with AXL levels and negatively correlated with expression levels of miR-33a-5p [2]. DANCR over-expression has also been detected in lung cancer, principally in high-grade samples and aggressive tumors [10]. Expression assays in hepatocellular cancer tissues have revealed over-expression of DANCR and ATG7, and down-regulation of miR-222-3p. Notably, DANCR levels have been positively correlated with poor clinical outcome in these patients [17]. Another study in hepatocellular carcinoma has shown up-regulation of DANCR in tumor and plasma samples in correlation with microvascular and hepatic capsule invasion. Most remarkably, plasma levels of DANCR have shown more appropriate discriminatory power for separation of patients with hepatocellular carcinoma from healthy controls and patients with chronic hepatitis B compared to α-fetoprotein [62]. In breast cancer samples, over-expression of DANCR has been associated with involvement of lymph nodes as well as hormone receptor and HER2 expressions [90]. Cumulatively, almost all studies in clinical samples have shown up-regulation of DANCR in malignant samples compared with their non-malignant counterparts. Exceptions to this rule are few studies in renal cell carcinoma [86], papillary thyroid cancer [91] and hepatocellular carcinoma [92]. Table 3 shows dysregulation of DANCR in clinical samples.

**Table 3:** Dysregulation of DANCR in clinical samples

Tumor type	Samples	Expression (tumor vs. normal)	Kaplan–Meier analysis (impact of DANCR dysregulation)	Univariate/multivariate cox regression	Association of dysregulation of DANCR with clinical data	References
Bladder cancer	120 PTN	Up	Shorter OS and DFS	High levels of DANCR were an independent prognostic factor for shorter OS	LN metastasis status, tumor stage, histological grade	[8]
	106 PTN	Up	–	–	Higher histological grade and advanced TNM stage	[9]
Breast cancer	TCGA dataset 60 triple-negative (TNBC) type, 15 HER2 type, 15 Luminal A type, and 15 Luminal B type, and 10 normal breast tissues	Up in TNBC	Shorter OS	–	Bigger tumor size	[27]
	30 PTN	Up	–	–	–	[28]
	TCGA database 46 PTN	Up	–	–	Advanced tumor grades or lymph node metastasis	[29]
	46 PTN	Up	–	–	–	[34]
	57 PTN	Up	Shorter OS	–	–	[35]
	35 TNBC tissues, 52 adjacent normal breast tissues and 25 non-TNBC breast tissues	Up-regulation of TUFT1(which induces DANCR expression) in TNBC tissues	Shorter OS	–	Lower differentiation degree of TNBC cells	[36]
	120 BC patients, 70 BBD patients, and 105 healthy controls	Up in BC patients	Shorter OS	DANCR was found to be an independent risk factor for BC	Lymph node metastasis, ER status, HER2 status, and TNM stage	[90]
	Five GEO datasets: 657 breast tumors 50 TNBC and 50 non-TNBC tissues	Up in TNBC tissues	–	–	OXC1/lnc-FOXCUT/lnc-DANCR axis is involved in the aggressive features of triple-negative breast tumors	[93]
	63 PTN	Up	Shorter OS	–	TNM stages	[37]
Breast cancer	TCGA database: 790 BCa tissues and 104 normal tissues 12 TNBC patients and 4 normal controls	Up	–	–	–	[38]
	2192 samples from 21 studies	Up	–	–	–	[94]
Cervical cancer	65 PTN	Up	Shorter OS	–	Advanced stage, larger tumors, advanced FIGO stage and lymph node metastasis	[39]
	82 PTN	Up	Shorter OS	–	Large tumor size, advanced FIGO stage	[40]
	112 PTN	Up	–	–	Histological type, tumor staging, infiltrating muscle depth and lymphatic metastasis	[41]
	33 PTN	Down-regulation of miR-665 (a target of DANCR) Up-regulation of DANCR	Shorter OS	–	Tumor size, distant metastasis, advanced TNM stage	[42]
Cholangiocarcinoma	40 PTN	Up	Shorter OS	–	Tumor size, TNM state and lymph node metastasis	[43]
	GEO database (GSE76297) 17 PTN	Up	–	–	–	[44]
Colon cancer	35 PTN	Up	–	–	–	[21]
	69 PTN	Up	Shorter OS	–	–	[22]
Colorectal cancer	50 PTN	Up	–	–	TNM stage and positive lymph node metastasis	[46]
	80 colorectal cancer patients and 10 normal colon tissues	Up	Shorter OS	–	Clinical stages	[47]
	40 PTN	Up	–	–	–	[48]
	GEO (GSE126092) and TCGA databases 15 PTN	Up	–	–	TNM stages	[95]
	47 PTN	Up	–	–	Clinical stage, nodal and metastasis classifications, and liver metastasis	[49]
Colorectal cancer	104 PTN	Up	Shorter OS and DFS	DANCR was found to be an independent poor prognostic factor for both OS and DFS	TNM stage, histologic grade, and lymph node metastasis	[96]
Endometrial carcinoma	27 patients and 18 normal controls	Up	–	–	–	[50]
Esophageal squamous cell carcinoma	51 PTN Data of KMPlot tool (55 patients) and data of LinkedOmics tool (178 patients)	Down-regulation of miR-33a-5p (a target of DANCR)	Shorter OS	–	Advanced TNM stage and lymph node metastasis	[52]
	32 PTN	Up	–	–	–	[53]
Gastric cancer	14 DDP-sensitive GC tissues and 14 DDP-resistant GC tissues	Up in DDP-resistant	–	–	–	[30]
	TCGA database 86 PTN	Up	Shorter OS	–	Tumor size, TNM stage, invasion depth, and lymph node metastasis	[31]
	65 PTN 55 patients and 39 healthy controls	Up	–	–	Tumor size, TNM stage, lymphatic metastasis and invasion depth	[32]
	118 PTN	Up	Shorter OS	–	–	[54]
Glioma	33 PTN	Up	Shorter OS	–	Clinical grading and tumor size	[55]
	TCGA dataset 82 glioma tissues and 10 normal brain tissues	Up	–	–	Tumor grading	[56]
	86 PTN	Up	Shorter OS	–	Histological type and WHO grade	[57]
	47 glioma patients and 14 normal tissues	Up	–	–	Advanced tumor grade	[59]
	TCGA database 40 tumor tissues and 40 normal tissues	Up-regulation of IGF2BP2 (which increases DANCR stability)	–	–	_	[61]
Hepatocellular carcinoma	62 PTN	Up	Shorter OS	–	–	[17]
	TCGA and GEPIA database 66 PTN	Up	Shorter OS	–	–	[18]
	13 HCC patients, 10 hepatitis, 10 with cirrhosis, and 10 normal database	Up in HCC patients	Shorter OS	High levels of DANCR were an independent prognostic factor	–	[19]
	STARBASE and GEPIA database	Up	Shorter OS	–	–	[20]
	52 PTN 52 HCC patients, 29 patients with chronic hepatitis, 22 cirrhosis and 43 healthy controls	Up in HCC patients	–	–	Microvascular and liver capsule invasion of HCC	[62]
	23 PTN	Down	–	–	–	[92]
Lung cancer	32 lung cancer tissues and 11 normal lung tissues	Up	Shorter OS		Grade	[10]
	GSE130779: 8 PTN 48 PTN	Up	Shorter OS		TNM stage and lymph node metastasis	[11]
	100 patients	Up	–		–	[13]
	34 PTN	Up	–		–	[12]
	45 PTN	Up	Shorter OS		Advanced TNM stage, lymph node metastasis and a larger tumor size	[63]
	TCGA database: lung 706 adenocarcinoma and 626 lung squamous cell carcinoma samples	Up	–		–	[14]
	40 PTN	Up	–		–	[64]
	64 PTN	Up	Shorter OS		Larger tumor size, advanced TNM stage and lymph node metastasis	[65]
	128 PTN	Up	–		–	[66]
	40 PTN	Up	–		–	[67]
Nasopharyngeal carcinoma	10 PTN 100 PTN	Up	Shorter OS	–	–	[70]
	14 tumor tissues and 9 normal tissues 212 tumor tissue	Up	Shorter OS and DFS and metastasis-free survival	DANCR expression and N stage were found to be independent prognostic factors	Lymph node metastasis	[73]
Oral squamous cell carcinoma	86 PTN	Up	Shorter OS	–	Histological grade, clinical staging and lymph node metastasis	[75]
Osteosarcoma	95 PTN	Up	Shorter OS		advanced stage, lymph node metastasis and distant metastasis	[3]
	34 PTN	Up	–		–	[2]
Osteosarcoma	109 PTN	Up	–		Lymph node metastasis and distant metastasis	[4]
	45 PTN	Up	–		–	[5]
	40 PTN	Up-regulation of METTL3 (DANCR could be a target of METTL3)	–	–	–	[6]
Ovarian cancer	20 PTN	Up	–	–	–	[78]
	20 PTN	Up	–	–	TNM staging and metastasis	[79]
Pancreatic cancer	30 PTN	Up	–	–	–	[24]
	50 PTN	Up	Shorter OS	–	Tumor size, TNM stage, and lymph nodal metastasis	[25]
	206 PTN	Up	Shorter OS and PFS	DANCR was found to be an independent poor prognostic factor for both OS and PFS	Vascular invasion, advanced T stage, lymph node metastasis and advanced TNM stage	[80]
	68 PTN	Up	–	–	TNM stage, N stage, and recurrence rates	[26]
Papillary thyroid cancer	GEO database (GSE33630, GSE50901, and GSE66783) 76 PTN	Down	–	DANCR was found to be an independent protective factor for TNM stage	TNM stage	[91]
	112 PTN	Up	–	–	Age and micro carcinoma	[97]
Prostate cancer	36 PTN	Up	–	–	–	[81]
	40 PTN	Up	Shorter OS	–	Grade and metastasis	[82]
	53 patients and 47 healthy controls	Up	Shorter OS	–	PSA, Gleason score, T stage, N stage and M stage	[83]
	15 DTX-sensitive and 14 DTX-resistant PC tissues	Up in DTX-resistant	–	–	–	[84]
	GEO database (GSE2547)	Up	–	–	–	[85]
Renal cell carcinoma	24 PTN	Down	–	–	–	[86]
Retinoblastoma	57 patients and matched health controls	Up	Shorter OS	–	–	[87]

OS: Overall survival, TNM: tumor node metastasis, TCGA: Cancer Genome Atlas, DFS: disease-free survival, HCC: hepatocellular carcinoma, PFS: progression-free survival, TNBC: Triple negative breast cancer, BC: breast cancer, BBD: benign breast disease, DTX: docetaxel, DDP: cisplatin, DFS: disease-free survival, PTN: pairs of tumor and normal samples

## Discussion

DANCR is regarded as an oncogene in almost all types of cancers. All conducted studies have indicated up-regulation of DANCR in cancer tissues/cell lines except for a single study in renal cell carcinoma [86]. Moreover, two studies in papillary thyroid cancer [91] and hepatocellular carcinoma [92] reported down-regulation of this lncRNA, in spite of the bulk of evidence regarding up-regulation of DANCR in these two types of cancers. In support of the oncogenic role of DANCR, several studies have indicated association between up-regulation of DANCR and poor clinical outcomes. Moreover, over-expression of DANCR has been more frequently detected in patients having advanced clinical stages and distant metastases.

Over-expression of DANCR has also been associated with resistance to anti-cancer agents such as cytarabine, sorafenib, cisplatin and docetaxel. These findings indicate that DANCR-targeting therapies might affect response of cancer cells to a wide array of drugs, possibly conquering multidrug resistance.

DANCR has also been shown to possess appropriate diagnostic power to differentiate patients with liver cancer from healthy persons or those with non-malignant liver disorders [62]. Since this expression assay has been conducted in plasma samples, it potentiates DANCR as a non-invasive marker for cancer detection.

Tens of tumor suppressor miRNAs have been shown to be sponged by DANCR, leading to release of miRNA targets from their inhibitory effects. DANCR can also regulate activity of several important cancer-related pathways such as PI3K/AKT/NF-κB, Wnt/β-catenin, ERK/SMAD, MAPK, IL-6/JAK1/STAT3, Smad2/3, p53, FAK/PI3K/AKT/GSK3β/Snail pathways. Since several signaling pathways are influenced by DANCR, drugs targeting this lncRNA are expected to affect numerous aspects of carcinogenesis, thus being effective in treatment of a wide range of cancers with different biological behaviors.

In addition, DANCR has interactions with a number of proteins including CTNNB1, RXRA, EZH2 and PRC2. Most importantly, interaction of DANCR with proteins that influence epigenetic marks shows the importance of DANCR in the regulation of gene expression.

## Conclusion

Although several expression assays have assessed expression levels of DANCR in biological samples obtained from different types of cancers, the underlying cause of dysregulation of DANCR in cancer has not been identified. In addition, the impacts of genomic variants on expression of this lncRNA and possible associations between single nucleotide polymorphisms within *DANCR* gene and susceptibility to cancer have not been appraised yet. Thus, future investigations should focus on these aspects. High throughput sequencing techniques could facilitate answering to these questions in near future.

## Data Availability

The analyzed data sets generated during the study are available from the corresponding author on reasonable request.
